# Prevalence and factors associated with low birth weight among newborns in South Sudan

**DOI:** 10.4314/ahs.v23i3.19

**Published:** 2023-09

**Authors:** Chol Lat, Florence Murila, Dalton Wamalwa

**Affiliations:** University of Nairobi College of Health Sciences, Department of Paediatrics and Child Health

**Keywords:** Low birth weight, improve birth outcomes, multivariate logistic regression analysis

## Abstract

**Background:**

WHO estimates that that 13% of babies are delivered low birth weight in Sub-Saharan Africa. Infants with LBW have a twenty times greater risk of dying than infants weighing more than 2500 grams. The neonatal mortality rates in South Sudan is 40 per 1000 live births. LBW significantly contributes to neonatal mortality rates.

**Objectives:**

The study aimed at determining the prevalence and factors associated with LBW among newborns.

**Methods:**

This was a cross-sectional descriptive study conducted at three hospitals. Completed data on all live births was collected using a structured questionnaire. Univariate and multivariate logistic regression analysis was applied for factors associated with LBW. Adjusted odds ratio with 95% confidence interval was applied and a P value <0.05 was considered statistically significant.

**Results:**

We retrieved records of 11845 birth cohorts. The prevalence of LBW among newborns was 11.4%. The prevalence of LBW at Aweil, Juba and Bor was 13.3%, 9.8% and 8.8% respectively. Maternal age less than 20 years and 35 years and above, multigravidity, GA <37 weeks, male sex and multiple pregnancy were significantly associated with LBW.

**Conclusion:**

The prevalence of LBW in infants was 11.4%. Associated factors were, maternal age, GA <37 weeks, multigravidity, male sex and multiple pregnancy.

## Introduction

Low birth weight (LBW) is defined by World Health Organization as weight at delivery of an infant less than 2500 grams. Prematurity is defined as delivery of an infant at less than 37 weeks gestation. Birth weight depends on gestational age and fetal growth during pregnancy.

The cut-off weight is centred on epidemiological interpretation that infants weighing less than 2,500 grams are approximately twenty times more likely to die compared to heavier babies 1.

Studies demonstrate that multiple influences such as maternal anemia and nutrition, parity and drug intake and smoking, socioeconomic status, obstetric complications, chronic illnesses, drug addiction and teenage pregnancy contribute to low birth weight. Approximately thirteen million infants are delivered before 37 completed weeks of gestation. This number can be significantly pronounced in middle-and low-income countries 2. Preterm delivery (<37 weeks) is highly associated with the development of respiratory distress syndrome (RDS), a lung condition occurring due to lack of surfactant. RDS can lead to significant infant morbidity and mortality.

In studies done by WHO and UNICEF globally, 15-25% newborn infants are delivered LBW. Twenty eight percent of the LBW infants are in South Asia, 13% are in Sub-Saharan Africa and 6% are in East Asia 3. Birth weight is a good indicator of community wellbeing as it reflects health care delivery at the community level and the socio-economic conditions of a given community, predicts neonatal viability and is inversely related to neonatal/perinatal mortality. South Sudan has one of the highest neonatal mortality rates in the world at 40 per 1000 live births 4 Neonatal mortality rates increase proportionally with decreasing gestational age or birth weight 5. The purpose of this study is to establish the prevalence and factors associated with low birth weight in South Sudan in the year 2018 using data from three hospitals (Juba Teaching Hospital, Aweil State Hospital and Bor State Hospital). The study results will provide information (there is no current available data) to stakeholders for informed decision-making in the allocation of resources to provide improved maternal and newborn services in the studied population.

## Methods

**Figure 1:**
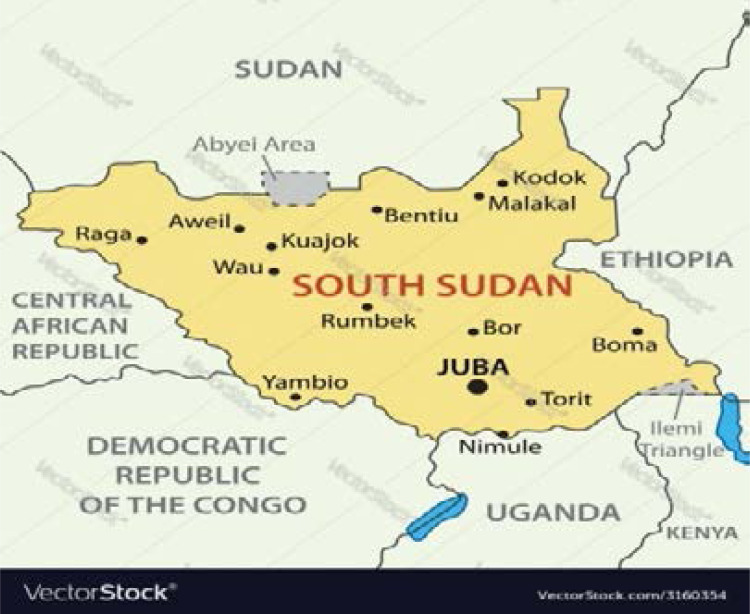
Map of South Sudan showing study locations: Juba, Aweil and Bor. Reproduced with permission from Vector Stock: https://www.vectorstock.com/royalty-free-vector/republic-south-sudan-map-vector-3160354

This was a hospital-based descriptive cross-sectional study which evaluated live birth cohorts of 2018 at three hospitals in South Sudan. After obtaining the ethical clearance from both University of Nairobi/ KNH (P570/07/2019) and South Sudan Ministry of Health (MOH/ERB52/2019), the selected hospitals in South Sudan were Juba Teaching, Aweil and Bor hospitals as shown in Figure 1. Using structured questionnaire, we collected maternal and newborn variables from the birth registry on each delivery within 24 hours of birth on all mothers who delivered and their newborns at the selected hospitals in 2018. Incomplete records and stillbirths were excluded. 11,845 mother-baby pairs met eligibility criteria. Data were entered in STATA version 15 for analysis. Univariate and multivariate logistic regression methods were applied to determine factors associated with LBW Adjusted odd ratios with 95% confidence interval was applied and a P value of less than 0.05 was considered statistically significant. Results were presented in form of tables, bar graphs, pie charts and proportions.

## Results

### Maternal demographic and clinical characteristics

We obtained 11845 maternal and infant records from the three facilities (Juba Teaching Hospital, Aweil State Hospital and Bor State Hospital). Out of the total deliveries, 4240 (35.8%) were from Juba Teaching Hospital, 5898 (49.8%) from Aweil State Hospital and 1707 (14.4 %) from Bor State hospital. Table 1 shows the maternal demographic and clinical characteristics of the study participants. Majority of the mothers were aged 20-34 years 9819(82.9%), 11647(98.33%) were HIV negative and 11375 (96.03%) had a singleton pregnancy.

**Table 1:** Maternal Demographic and Clinical Characteristics (N=11845)

Characteristic	Frequency	Percent
**Maternal Age**		
Less thnn 200 yrs.	1,453	12.3
20-34 yrs.	9819	82.9
Above 35 yrs.	573	4.8
**Gravidity**		
Primigravida	2,652	22.4
Multigravida	9193	77.6
**HIV status**		
Negative	11,647	98.3
Positive	198	1.7
**Birth Plurality**		
No	11,375	96.0
Yes	470	4.0

### Infant Characteristics

The majority of the births were male babies 6146 (51.9%) and 10307 (87%) weighed between 2.5 and 4kgs. Most of the babies 9867 (83.3%) were term, 11124 (93.9%) recorded an APGAR score of 7-10 and 11784 (99.49%) did not have congenital anomalies. Table 2 shows a summary of infant characteristics of the study participants.

**Table 2:** Infant Characteristics (N=11845)

Characteristic	Frequency	Percent
Gender		
Female	5,699	48.1
Male	6,146	51.9
Birth Weight less than 1kg	47	0.4
1-<1.5kgs	124	1.1
1.5-<2.5kgs	1,184	10
2.5-4kgs	10,307	87.0
Above 4kgs	183	1.5
APGAR score		
0-3	272	2.3
4-6	449	3.8
7-10	11,124	93.9
Congenital anomaly		
No	11,784	99.5
Yes	61	0.5
Gestational Age less than 28weeks	77	0.7
28- <32 weeks	255	2.2
32- <37 weeks	1,571	13.2
37- 42 weeks	9,867	83.3
42 weeks and above	75	0.6

### Prevalence of low birth weight

**Figure 2:**
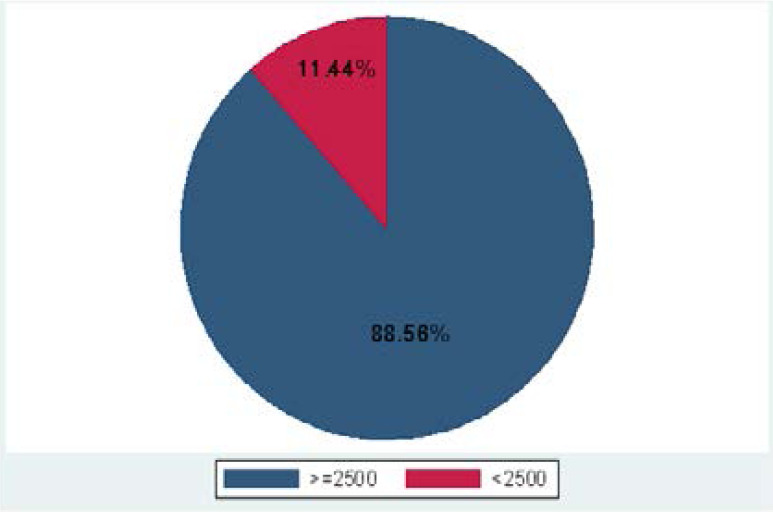
The overall prevalence of low birth weight in the three selected hospitals

**Figure 3:**
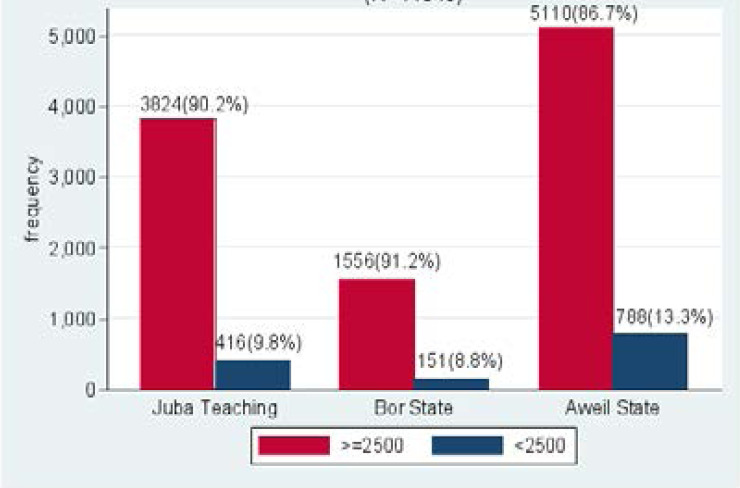
Prevalence of low birth weight in each of the hospitals; Juba, Bor and Aweil

The overall prevalence of low birth weight in the study population was 1355(11.4%), ranging from 8.8% at Bor State Hospital to 9.8% at Juba Teaching Hospital, to 13.3% at Aweil State Hospital (as shown in Figures 2 and 3).

### The proportion of birth weight relative to gestational age

**Figure 4:**
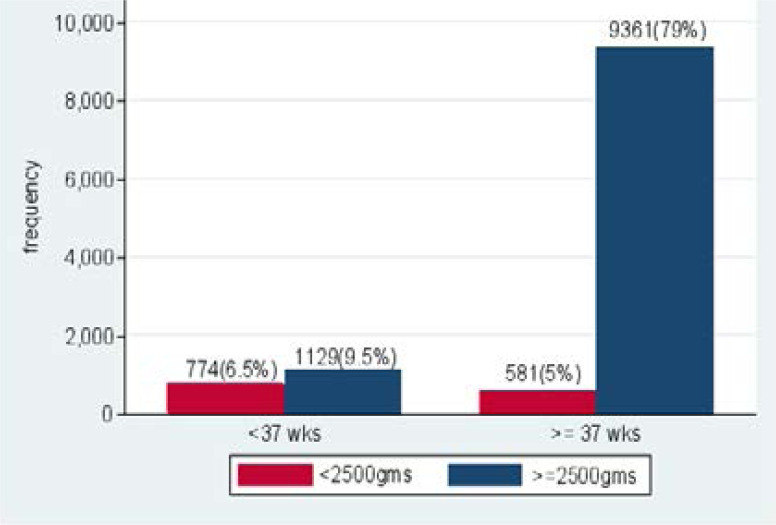
The proportion of birth weight relative to gestational age

Figure 4 shows the proportion of birth weight for gestational age in Juba, Bor and Aweil hospitals. Sixteen percent of the newborns were preterm compared to 9942(84%) delivered at 37 weeks and above. There were 774 (6.5%) preterm babies weighed less than 2500gms. Among the low-birth-weight infants 42.8% were term infants, indicating that a significant number of low birth deliveries could be as a result of intrauterine growth restriction.

### Factors associated with low birth weight

In the study we explored maternal and fetal factors associated with low birth weight. We used univariate and multivariate logistic regression to describe factors associated with low birth among the newborns. In the univariate analysis as shown in table 3, maternal age less than 20 years (OR=2.5, 95% CI: 2.17-2.88, p=0.000), multigravidity (OR=0.49, 95% CI: 0.44-0.56, p=0.000), gestational age less than 37 weeks (OR=11, 95% CI: 9.812.5, p=0.000), male babies (OR=2.5, 95% CI: 2.17-2.88, p=0.000) and multiple pregnancy (OR=27.47, 95% CI: 22.16-34.8, p=0.000) were significantly associated with low birth weight.

**Table 3:** Univariate model for factors associated with low birth weight (N = 11845)

Characteristic	≥2500gms (%)	<2500gms (%)	COR (95% CI)	P value
**Maternal Age**				
20-34 years	8865 (84.5)	954 (70.4)	1(Ref)	
<20 years	1145 (10.9)	308 (22.7)	2.5(2.17, 2.88)	**0.000**
Above 35 years	480 (4.6)	93 (6.9)	1.8 (1.4, 2.3)	
**Gravidity**				
Primigravida	2172 (20.7)	469 (34.6)	1(Ref)	
Multigravida	8318 (79.3)	886 (65.4)	0.49(0.44, 0.56)	**0.000**
**Gestational Age**				
≥ 37 weeks	9361 (89.2)	581 (42.9)	1(Ref)	
<37 weeks	1129 (10.8)	774 (57.1)	11 (9.8, 12.5)	**0.000**
**Gender of the baby**				
Female	4,959 (47.3)	740 (54.6)	1(Ref)	
Male	5,531 (52.7)	615 (45.4)	0.75(0.67, 0.84)	**0.000**
**Mother's HIV status**				
Negative	10,311 (98.3)	1,336 (98.6)	1(Ref)	
Positive	179 (1.7)	19 (1.4)	1.26(0.74, 2.15)	**0.389**
**Congenital Anomaly**				
No	10,435 (99.5)	1,349 (99.6)	1(Ref)	
Yes	55 (0.5)	6 (0.4)	0.93(0.40, 2.17)	**0.859**
**Birth Plurality**				
Singleton	10,365 (98.8)	1,010 (74.5)	1(Ref)	
Multiple	125 (1.2)	345 (25.5)	27.47(22.16,34.05)	**0.000**

Table 4 shows logistic regression model for factors associated with low birth weight. Findings indicate that maternal age less than 20 years (aOR=1.59, 95% CI: 1.29-1.96, p=0.000) and those aged 35 years and above (aOR=1.70, 95% CI: 1.28-2.27, p=0.000), multigravidity (aOR=0.62, 95% CI: 0.52-0.75, p=0.000), gestational age less than 37 weeks (aOR=12.33, 95% CI: 10.7-14.2, p=0.000), male babies (aOR=0.75, 95% CI: 0.66-0.86, p=0.000) and multiple pregnancy (aOR=41.44 95% CI: 32.57-52.72, p=0.000) were significantly associated with low birth weight.

**Table 4:** Multivariable model for factors associated with low birth weight (N= 11845)

Characteristics	≥ 2500gms (%)	<2500gms (%)	AOR (95% CI)	P value
**Maternal Age**				
20-34years	8865 (84.5)	954 (70.4)	1 (Ref)	
<20years	1145 (10.9)	308 (22.7)	1.59 (1.29, 1.96)	**0.000**
35 years and above	480 (4.6)	93 (6.9)	1.70 (1.28, 2.27)	
**Gravidity**				**0.000**
Primigravida	2172 (20.7)	469 (34.6)	1 (Ref)	
Multigravida	8318 (79.3)	886 (65.4)	0.62 (0.52, 0.75)	**0.000**
**Mother's HIV status**				
Negative	9361 (89.2)	581 (42.9)	1 (Ref)	
Positive	1129 (10.8)	774 (57.1)	0.64 (0.37, 1.1)	**0.108**
**Gestational Age**				
≥ 37 weeks	4,959 (47.3)	740 (54.6)	1 (Ref)	
<37 weeks	5,531 (52.7)	615 (45.4)	12.33 (10.7, 14.2))	**0.000**
**Baby's gender**				
Female	10,311 (98.3)	1,336 (98.6)	1 (Ref)	
Male	179 (1.7)	19 (1.4)	0.75 (0.66, 0.86)	**0.000**
**Congenital Anomaly**				
No	10,435 (99.5)	1,349 (99.6)	1 (Ref)	
Yes	55 (0.5)	6 (0.4)	0.63 (0.25, 1.57)	**0.316**
**Birth Plurality**				
Singleton	10,365 (98.8)	1,010 (74.5)	1 (Ref)	
Multiple	125 (1.2)	345 (25.5)	41.44(32.57,52.72)	**0.000**

## Discussion

This report is a descriptive cross-sectional study in three selected hospitals of South Sudan. The main objectives of this study were to determine the prevalence and factors associated with low birth weight among newborns in the selected hospitals of South Sudan.

In the three hospitals, the overall prevalence of LBW was observed to be 11.4%. Aweil State Hospital, Juba Teaching Hospital and Bor State Hospital recorded LBW prevalence of 13.3%, 9.8% and 8.8%, respectively. Aweil State Hospital recorded the highest prevalence of LBW compared to the other two hospitals. This could be due to the social-demographic characteristics of the population or the inability of organizations and the capacity of health systems to meet needs in the period of antenatal care. Findings in Aweil State Hospital are consistent with the findings by UNICEF and WHO in Sub-Saharan Africa. In the studies done by WHO and UNICEF, it has been shown that 15-25% of neonates are LBW globally, of which (28%) of LBW were recorded in South Asia and 13% in sub-Saharan Africa^3^.

In a Nigerian study in 2015, by Henry et al in a Traditional Birth Home in the city of Benin, the prevalence of low birth was 6.3%, two times lower than our study, although closer to the findings at Bor State Hospital (8.8%). In the Nigerian study, gestational age and maternal age were significantly associated with low birth weight^6^. Our study also demonstrated that gestational age and maternal age are significantly associated with low birth weight.

The overall prevalence of low birth weight of 11.4% in this study is lower compared to a study by Saeed O et al in Khartoum State,13% 7. Similar to Saeed O et al study, our findings also demonstrated that gestational age and birth plurality were significantly associated with low birth weight. A study in Malaysia by Kaur et al in 2019 demonstrated that rural women had more low birth weight babies than urban women^8^. Similarly, a study by Demelash et al in one region of Ethiopia showed that women residing in rural areas were two times more likely to deliver LBW babies than their urban counterparts^9^.

Our study found that 42.8% of low birth weight were term infants. This indicates that a significant number of low-birth-weight infants may be experiencing IUGR. A similar study done by G. Donzeli et al at Nkubu Rural Hospital in Kenya showed that the incidence of low birth weight was 7% of which 79.6% were term, small for gestation 10. A study by Villar et al showed that most low-birth-weight infants in developed countries are born preterm while in developing countries about 75% of instances of low birth weight are delivered small for gestation due to intrauterine growth restrictions 11. The study by Villar et al shows that the incidence of IUGR (small for gestational age) is decreasing in Sub-Saharan Africa from 75% in 1982 to 25% in 2000. The higher prevalence of small for gestation at 42.8% in our study could be a result of socioeconomic factors in these communities.

The highest rates of preterm birth are in Sub-Saharan Africa and Asia which account for half of the world's births and more than 60% of the world's preterm babies in our study, the proportion of preterm infants was 16% (1903) of the total deliveries. In 2015, USAID estimated the prevalence of prematurity in South Sudan at 13% 12. Neither our study nor the USAID study was multi-center or representative of the whole country. However, the finding of our study is similar to a study done in Kenya by Peter Wagura et al which showed that the prevalence of preterm delivery was 18.3% 13. It is important to note that the majority of countries do not have reliable information on premature deliveries. WHO estimates that 3 million neonates die yearly, of which 1 million deaths are directly related to prematurity Reducing deaths related to prematurity and low birth weight can lead to the achievement of Sustainable Development Goal 3 (SDG 3) of reducing neonatal mortality to at least as low as 12 per 1000 live births by 2030. Since our study presents the prevalence of prematurity in just the three hospitals, it would be an overstatement if we project the findings as representative of the whole country. A better understanding of the causes of preterm birth and improved estimates of the incidence of preterm birth at the country level are needed in order to improve access to effective obstetric and neonatal care.

In our study maternal age less than 20 years or 35 years and above was significantly associated with low birth weight. A study by Razia Shaheen et al found similar results in their Bangladesh, Dhaka study: among the adolescent mothers, the low birth weight was 41%, while in mothers aged between 20-35 years, the rate was 28%, and among mothers age > 36 years the rate was 26.7%; hence there was a significant association between birth weight and maternal age^14^. A study by Alison et al in Utah, USA showed that despite adjusting for socio-economic factors which were thought to influence the prevalence of low birth weight in the mothers of all age groups, there still appeared to be an attributable significant role of biological factors intrinsic to maternal youth which conferred an increased risk of adverse pregnancy outcomes that was independent of important confounding sociodemographic factors 15. Another study in the United States by Xi-Kuan et al suggests that teenage pregnancy increases the risk of adverse birth outcomes that is independent of known confounding factors 16. Although no studies have been done to determine the incidence of teenage pregnancies and marriage, the South Sudan Child Act 2008 advocates for prohibition of teenage marriage and pregnancy. The strategies for prevention of teenage marriage and pregnancy include promotion of girl-child education and fines for those guardians who force their children to marry at an early age.

Our study showed that maternal age 35 years or more is associated with low birth weight. In contrast, the study by Razia Shaheen et al in Dhaka found that the risk of delivering low birth weight babies decreased with maternal age above 35 years and was lower compared to teenage mothers 14. The high rate of preterm birth could be related to complicated pregnancies that are more common in older mothers. In these older women, medical conditions such as, gestational diabetes, placenta praevia, breech presentation, could be the cause of LBW deliveries. These findings are consistent with those of some previous studies conducted in developing countries, studies by USAID and Rezende and colleagues 17,12.

Our study demonstrated that gestational age was associated with low birth weight and this was statistically significant. Gestational age <37 weeks was associated with low birth weight and statistically significant. Among the low birth weight infants, 57.2% were delivered before 37 weeks of gestation. A study by Apoorva et al at Hyderabad city, an urban setting in India observed similar results as 49.3% of low-birth-weight babies were delivered preterm 18.

In our study, being male was statistically significantly associated with low birth weight. However, an Ethiopian study by Mengesh H et al showed that female infants had higher chances of low-birth-weight delivery compared to male infants 19.

Our study also demonstrated that multiple gestation was statistically significantly associated with low birth weight. Women who had multiple pregnancy had a higher chance of delivering low birth weight babies. Multiple gestation is considered a high-risk pregnancy as it is associated with medical conditions such as high blood pressure during pregnancy, intrauterine growth restriction, early detachment of placenta, among others which can lead to premature delivery, hence low birth weight babies. A study in Nigeria by Dahlui et al using multiple logistic regression analysis also demonstrated that twin pregnancy was significantly associated with low birth weight 20. Although multiple pregnancy is not a modifiable factor in order to reduce the incidence of low birth, medical conditions that may decrease premature delivery in mothers with multiple gestation should appropriately be identified and managed.

In our study, using multivariable logistic regression analysis revealed multigravidity was indirectly associated with low birth weight. Multigravida were 38% less likely to deliver low birth weight babies. A study in Ethiopia by M. Mekie et al showed that being a multigravida had low risk of delivering low birth weight infant, a finding similar to our study. The number of previous pregnancies and deliveries influence the risk associated with the current pregnancy 19.

Although most of the findings associated with low birth weight are non-modifiable, mitigating risk factors associated with low birth can ensure a lot of cost-saving both to the government and the public.

The limitations in our study were incomplete birth records, not all deliveries in South Sudan occur in hospitals and still-births not being weighed. It is the first study in the country to explore the profile of LBW and establish base-line data which could be used by authorities to inform decisions to improve healthcare delivery in the communities.

## Conclusion

This study established the prevalence of low birth weight at 11.4% and factors associated with low birth weight, such as maternal age less than 20 years and age 35 years and above, gestational age less than 37 weeks, and multiple pregnancy were significantly associated with low birth weight. The findings are similar to those in other studies in the region. Policy-makers could use the outcome of these studies to improve health service delivery with the view to reducing low birth weight and potentially reduce neonatal mortality.
